# Supplementary material for: Modelling of dysregulated glucagon secretion in type 2 diabetes by considering mitochondrial alterations in pancreatic α-cells
Source: R Soc Open Sci. 2020 Jan 22;7(1):191171. doi: 10.1098/rsos.191171 (PMC7029933; doi:10.1098/rsos.191171)
Supplement: Supplementary Information [file rsos191171supp1.docx]

**Supplemental Information**

Modelling of dysregulated glucagon secretion in

type 2 diabetes by considering mitochondrial

alterations in pancreatic alpha cells

Vladimir Grubelnik, Rene Markovič, Saška Lipovšek, Gerd Leitinger, Marko Gosak, Jurij Dolenšek, Ismael Valladolid-Acebes, Per-Olof Berggren, Andraž Stožer, Matjaž Perc, Marko Marhl

**Supplementary Table**

**Table S1** Body weight, blood glucose, and serum insulin levels in mice (N = 6 per group) fed either the control or the western diet at the end of the diet intervention. *** denotes statistically significant differences, p<0.001 (t-test).

|  | Body weight (g) | Blood glucose (mmol/L) | Serum Insulin (ng/mL) |
| --- | --- | --- | --- |
| Control diet | 31.3 ± 0.79 | 7.53 ± 0.33 | 0.57 ± 0.03 |
| Western Diet | 40.73 ± 1.12*** | 10.03 ± 0.33*** | 2.62 ± 0.27*** |

**S1 Supplementary EM images**

**S1.1. Western Diet**


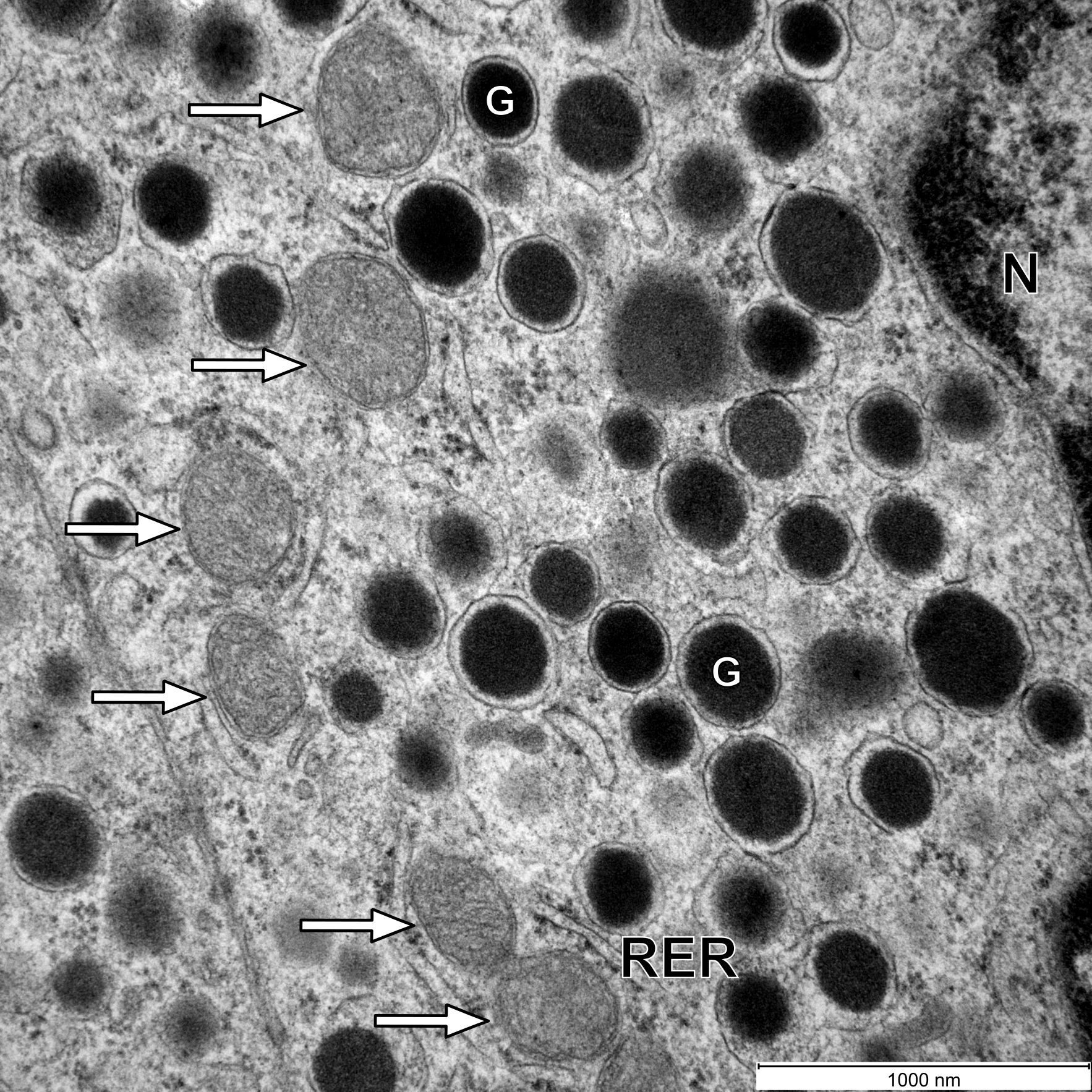


**Fig. S1** Ultrastructure of an α-cell in a WD-fed mouse. Mitochondria are round, have a less expressed matrix, and a decreased number of cristae. G, glucagon granule; N, nucleus; RER, rough endoplasmic reticulum; the white arrows point on mitochondria. Scale bar: 1000 nm.


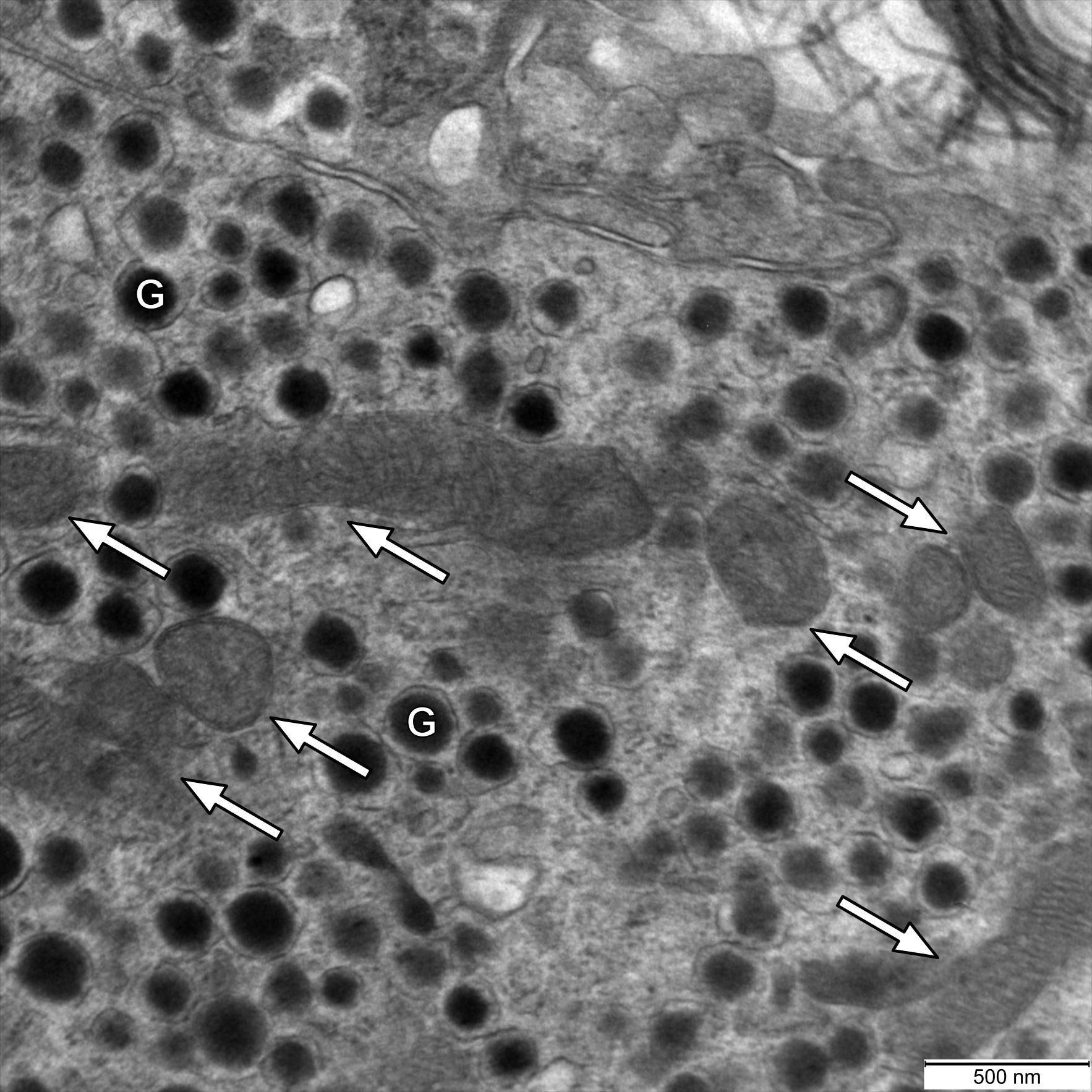


**Fig. S2** Ultrastructure of an α-cell in a WD-fed mouse. Mitochondria have a less expressed matrix and a decreased number of cristae. G, glucagon granule; the white arrows point on mitochondria. Scale bar: 500 nm.


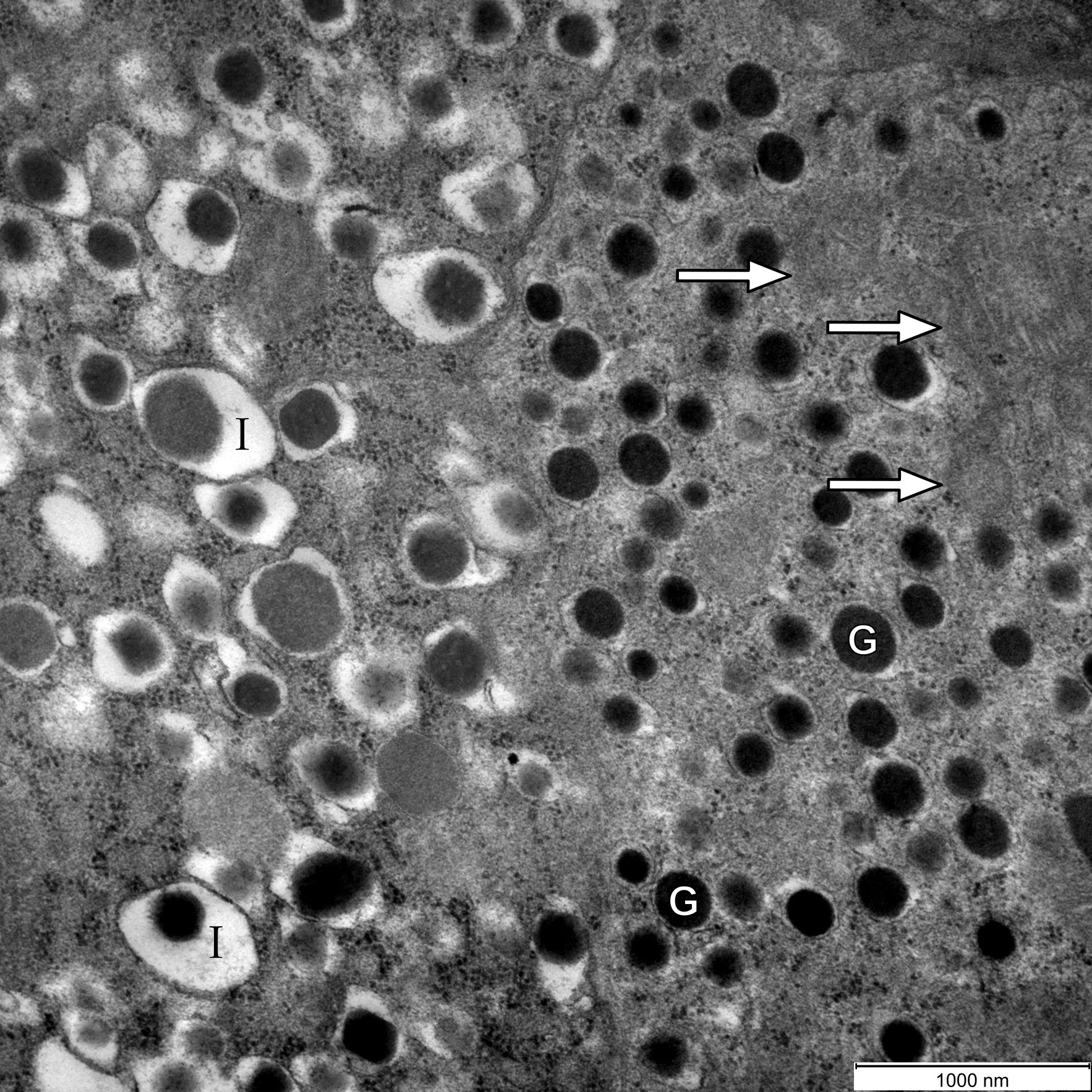


**Fig. S3** Ultrastructure of an α- and a $\beta$-cell in a WD-fed mouse. Mitochondria in the α-cell are round, have a less expressed matrix, and a decreased number of cristae. G, glucagon granule; I, insulin granule; the white arrows point on mitochondria. Scale bar: 1000 nm.


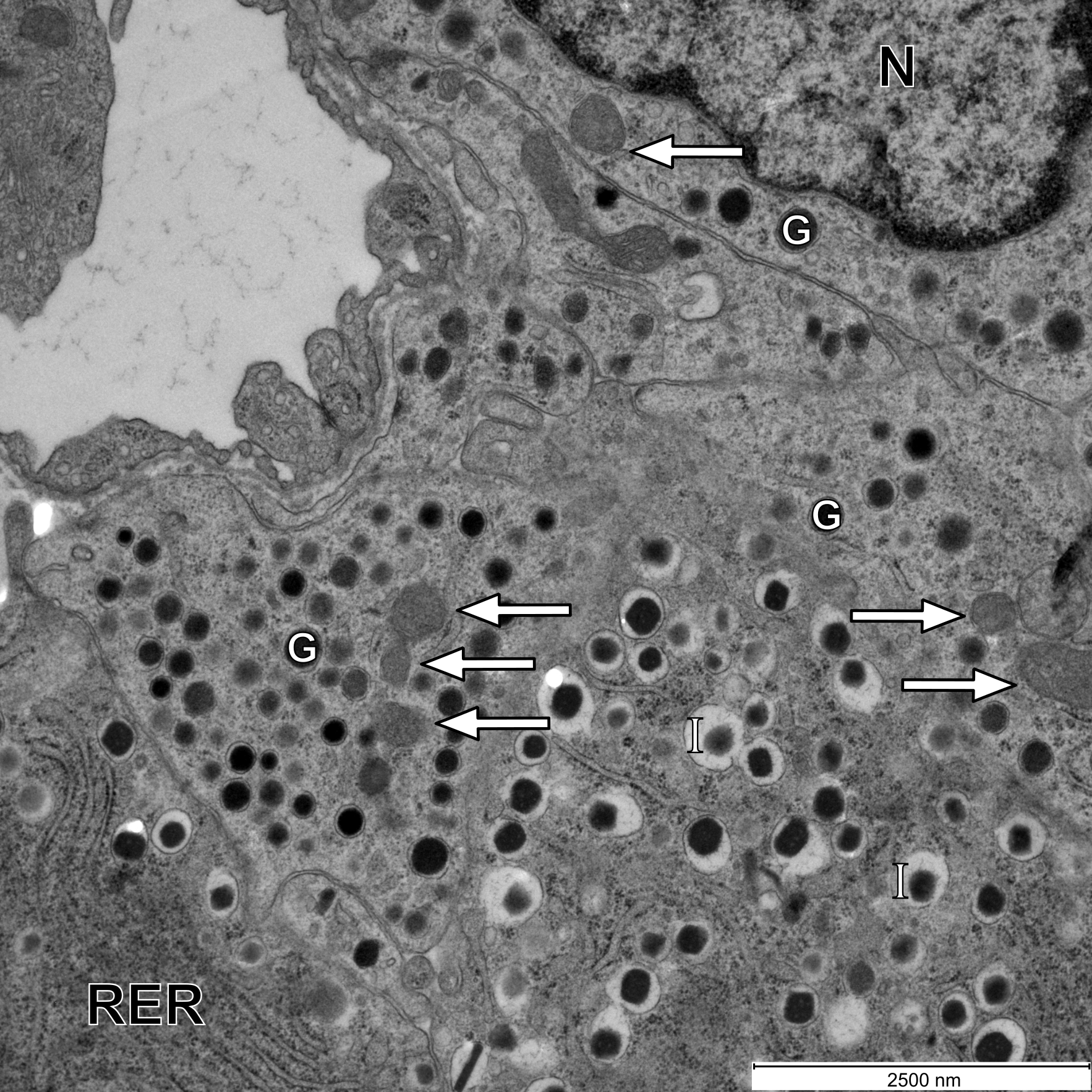


**Fig. S4** Ultrastructure of an α- and a $\beta$-cell in a WD-fed mouse. Mitochondria in the α-cell have a less expressed matrix and a decreased number of cristae. G, glucagon granule; I, insulin granule; N, nucleus; RER, rough endoplasmic reticulum; the white arrows point on mitochondria. Scale bar: 2500 nm.


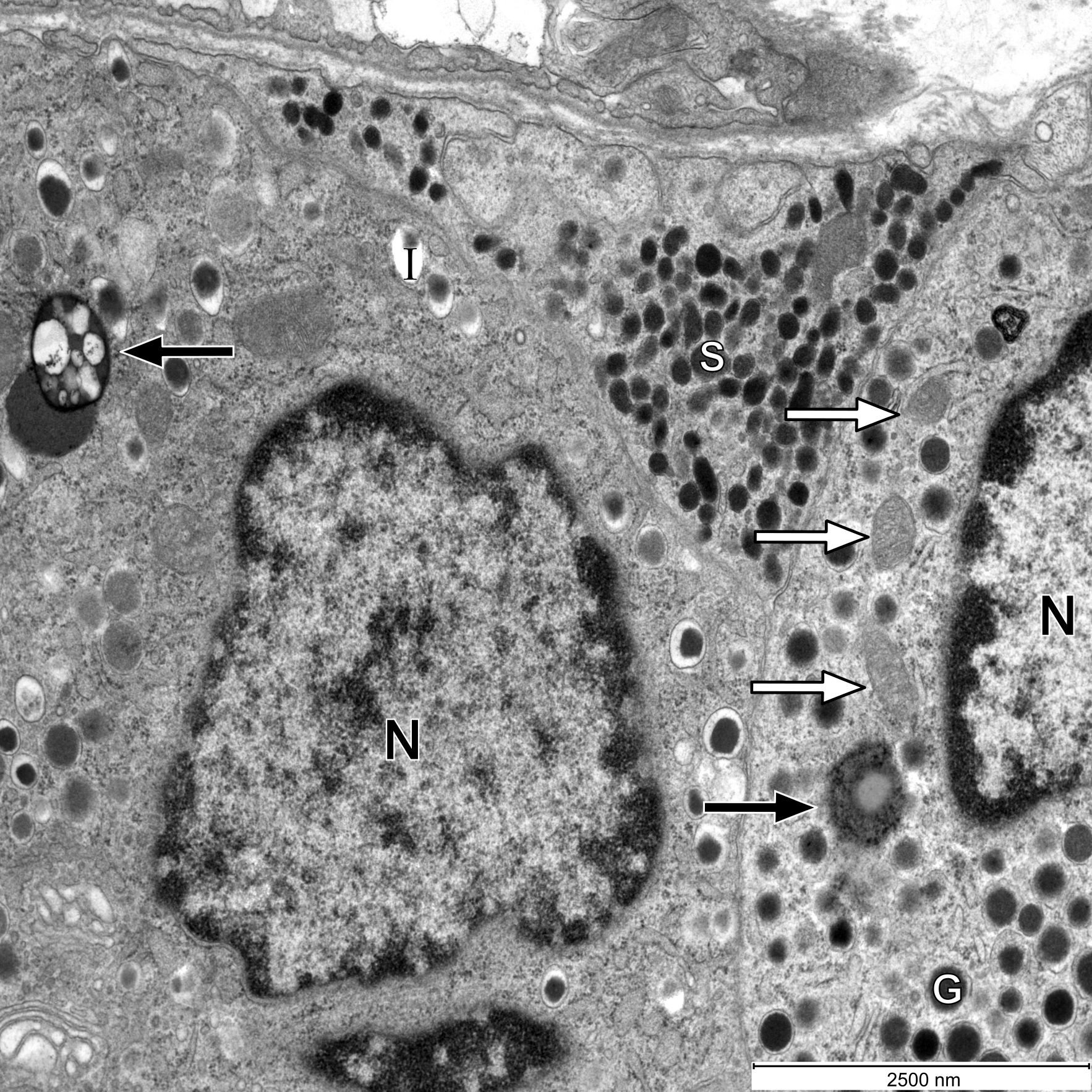


**Fig. S5** Ultrastructure of an α-, a $\beta$-, and a $\delta$-cell in a WD-fed mouse. Mitochondria in the α-cell have a less expressed matrix and a decreased number of cristae. In the α- and $\beta$-cell, one autolysosome is present in each cell. G, glucagon granule; I, insulin granule; N, nucleus; RER, rough endoplasmic reticulum; S, somatostatin granule; the white arrows point on mitochondria; the black arrows point on autolysosomes. Scale bar: 2500 nm.


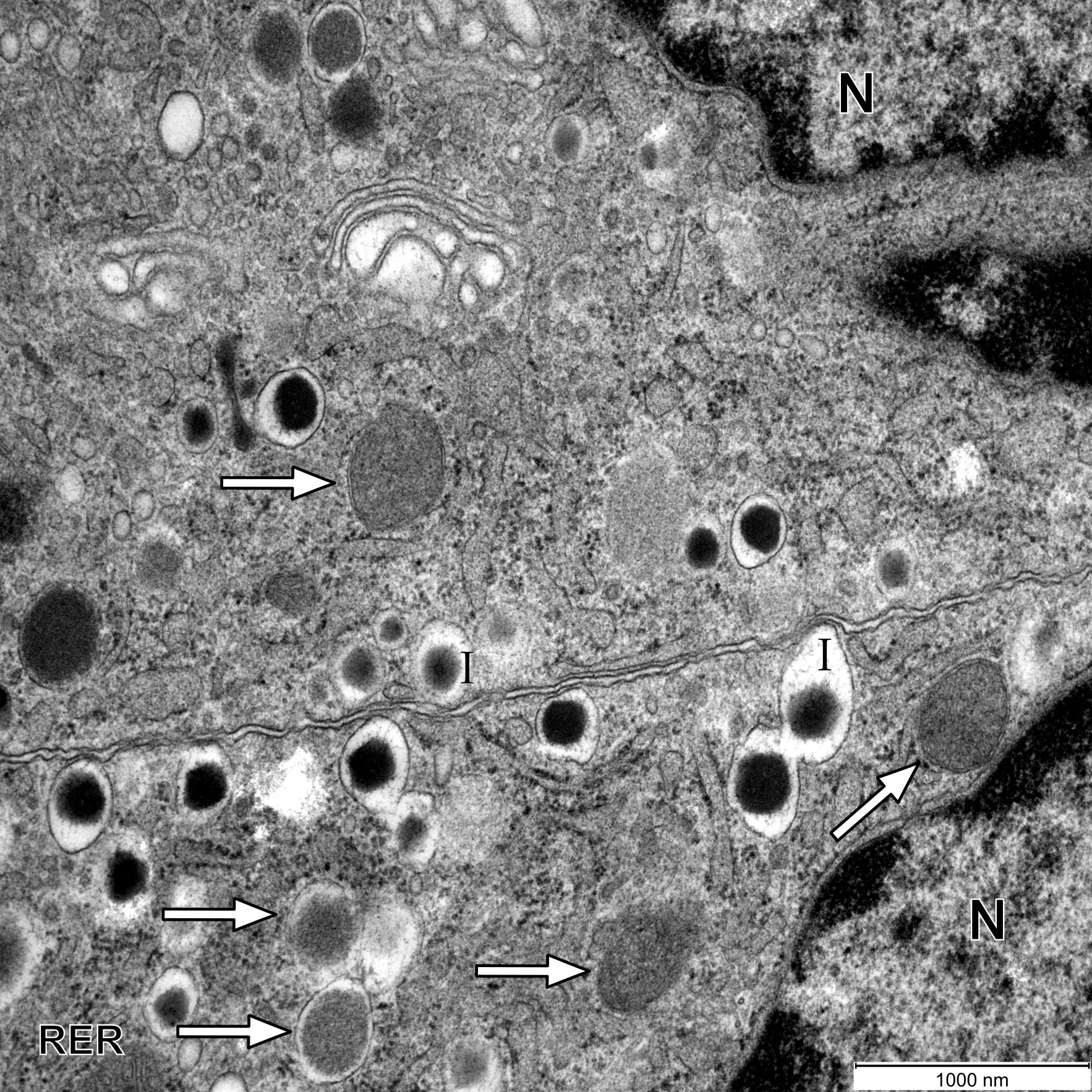


**Fig. S6** Ultrastructure of $\beta$-cells in a WD-fed mouse. Mitochondria are round, have a less expressed matrix, and a decreased number of cristae. I, insulin granule; N, nucleus; RER, rough endoplasmic reticulum; the white arrows point on mitochondria. Scale bar: 1000 nm.


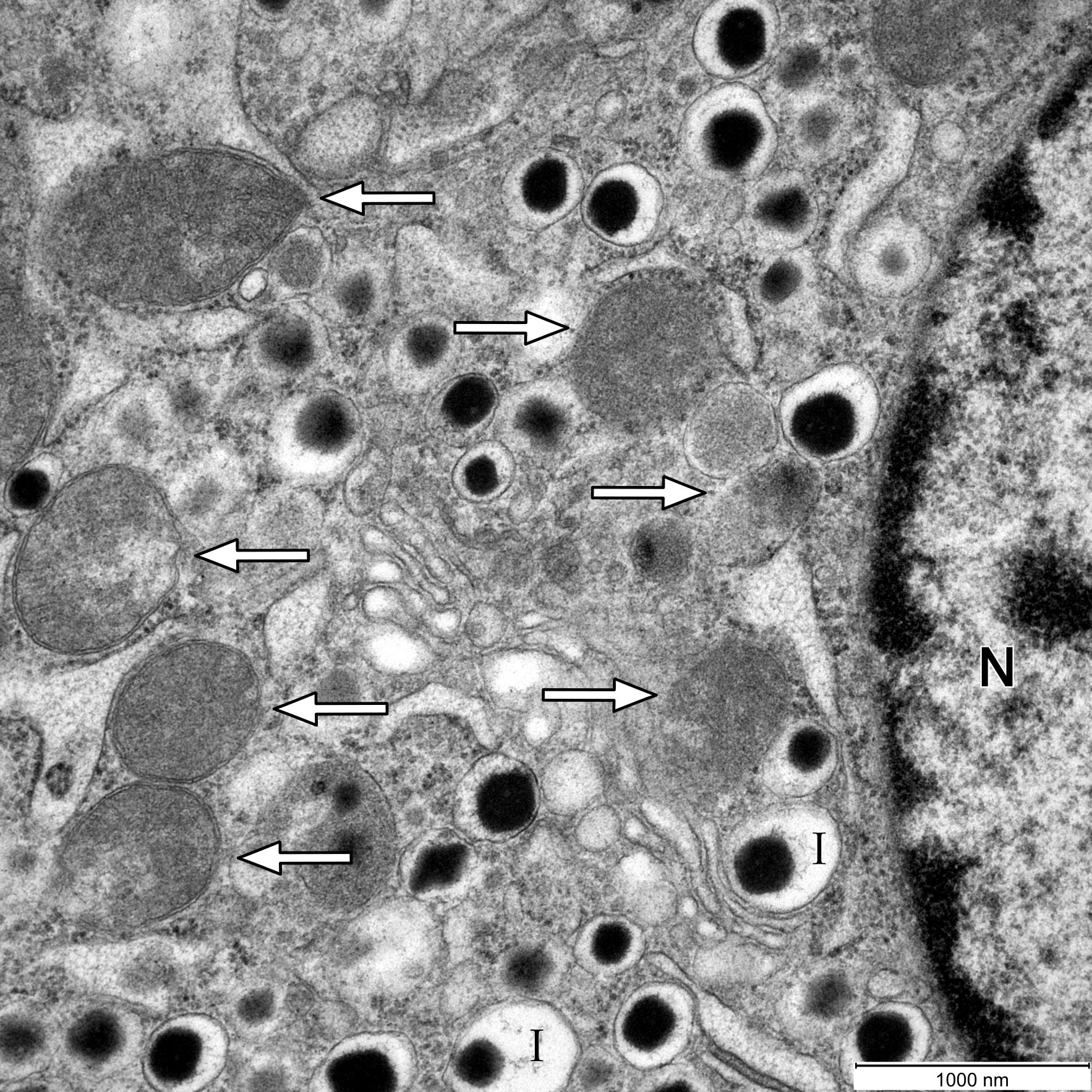


**Fig. S7** Ultrastructure of a $\beta$-cell in a WD-fed mouse. Mitochondria are round, have a less expressed matrix, and a decreased number of cristae. I, insulin granule; N, nucleus; the white arrows point on mitochondria. Scale bar: 1000 nm.


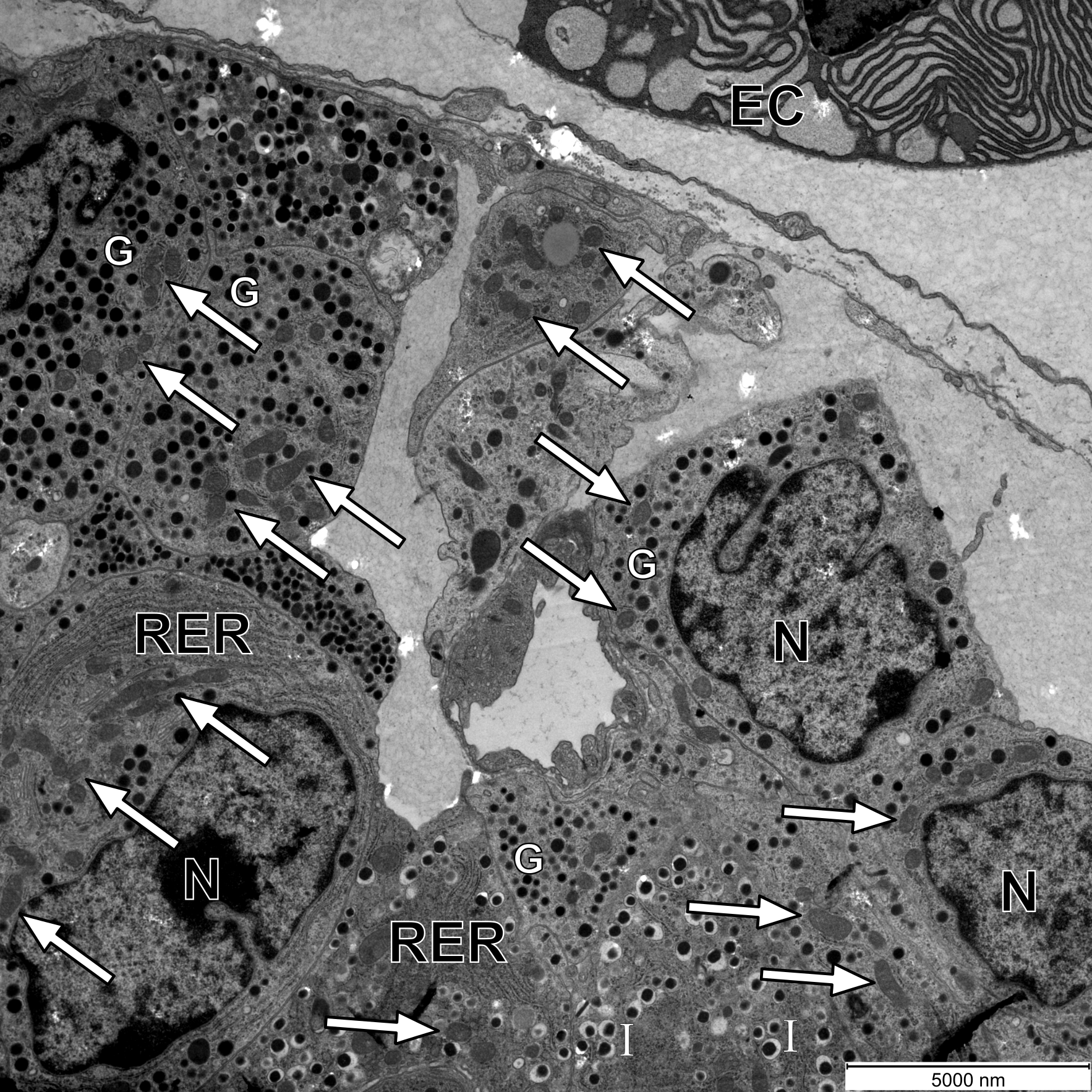


**Fig. S8** Ultrastructure of α- and $\beta$-cells in a WD-fed mouse. The majority of mitochondria are round, have a less expressed matrix, and a decreased number of cristae. In the upper part of the figure, an exocrine cell (EC) of the pancreas can also be seen. G, glucagon granule; I, insulin granule; N, nucleus; RER, rough endoplasmic reticulum; the white arrows point on mitochondria. Scale bar: 5000 nm.

**S1.2 Control**


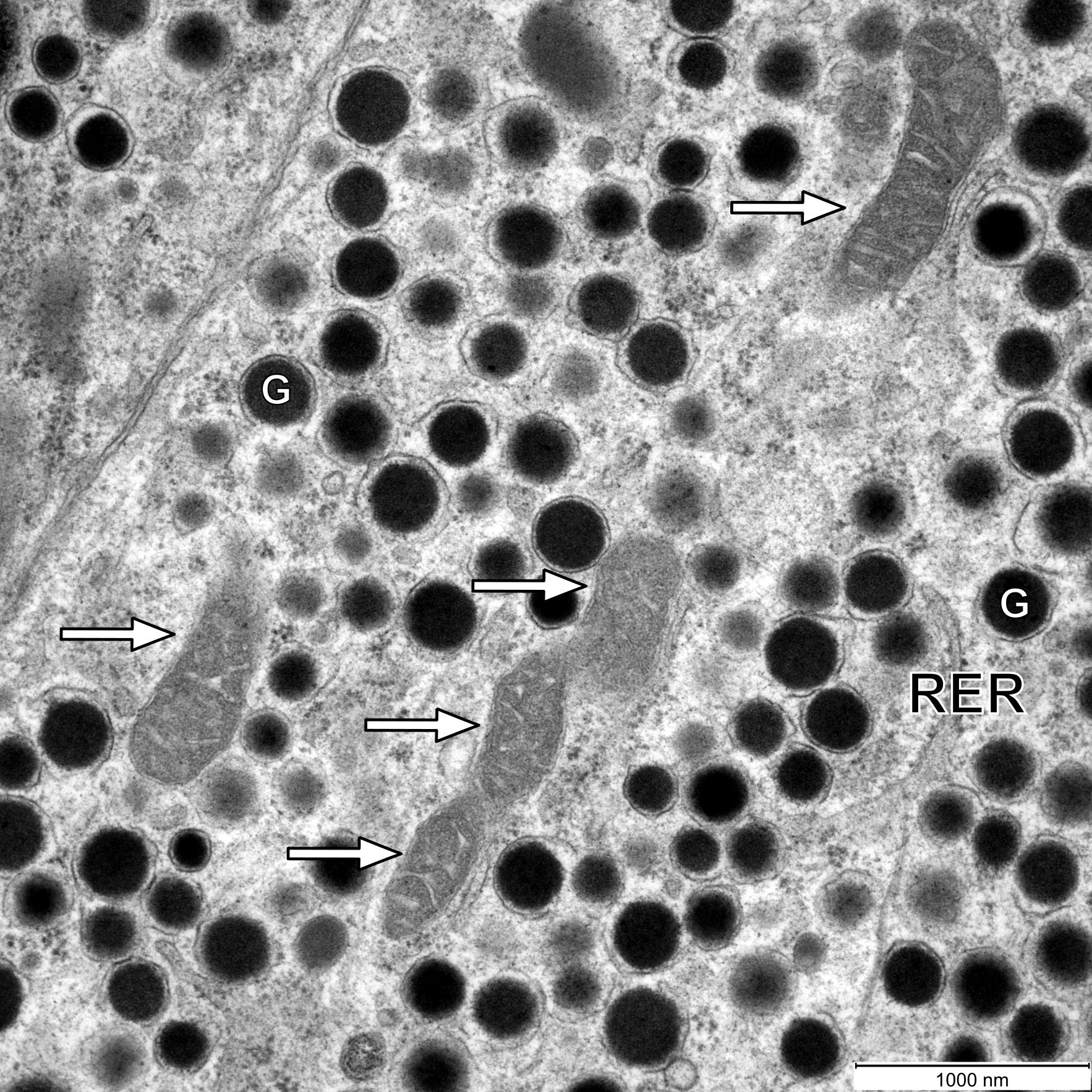


**Fig. S9** Ultrastructure of an α-cell in a control mouse. The cell contains numerous glucagon granules, well-structured mitochondria, and rough endoplasmic reticulum. All mitochondria are elongated, oval, showing the typical inner organization. The outer and inner membranes are well seen and the inner membrane is organized in characteristic cristae. G, glucagon granule; RER, rough endoplasmic reticulum; the white arrows point on mitochondria. Scale bar: 1000 nm.


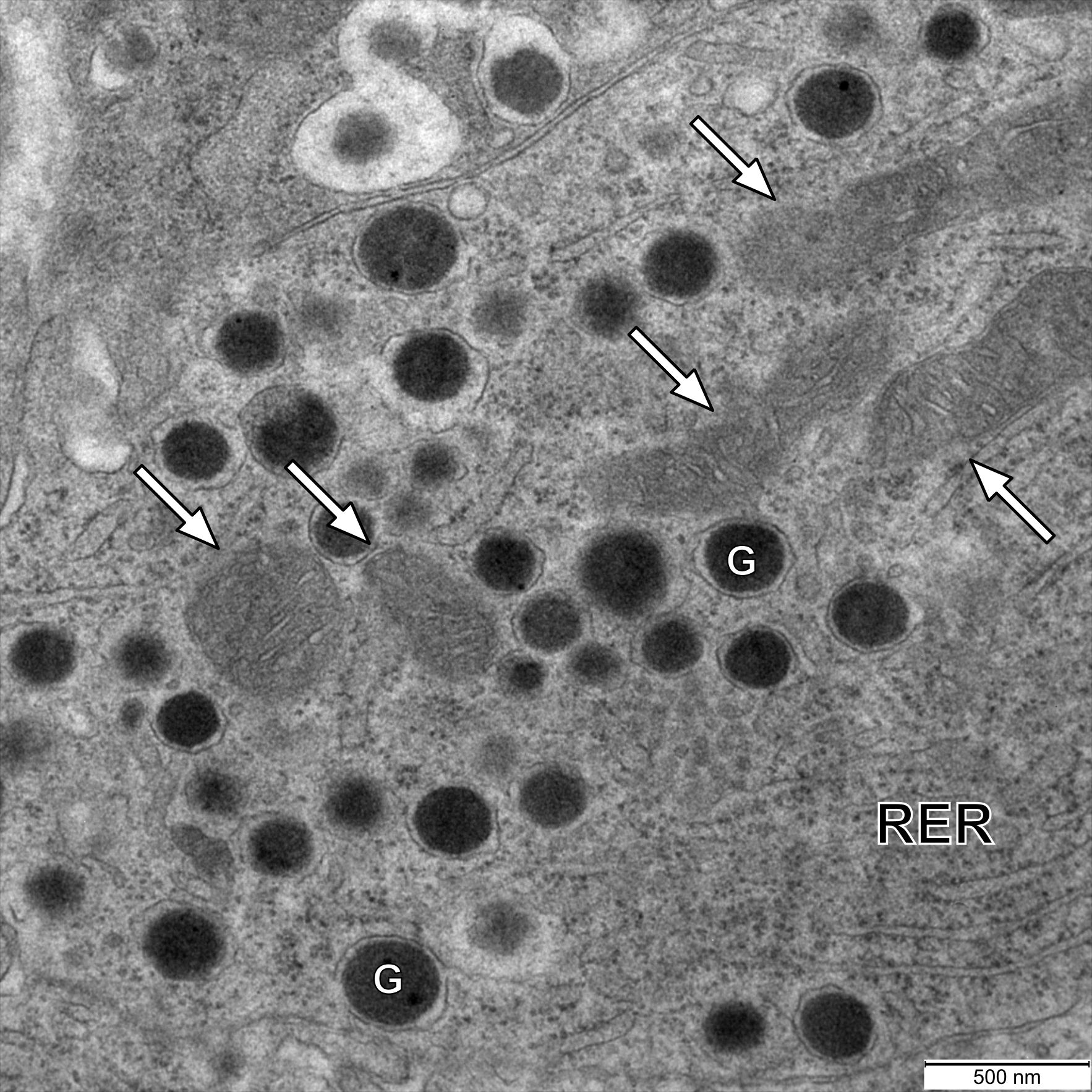


**Fig. S10** Ultrastructure of an α-cell in a control mouse. The cell contains numerous glucagon granules, well-structured mitochondria, and rough endoplasmic reticulum. All mitochondria are elongated, oval, showing the typical inner organization. The outer and inner membranes are well seen and the inner membrane is organized in characteristic cristae. G, glucagon granule; RER, rough endoplasmic reticulum; the white arrows point on mitochondria. Scale bar: 500 nm.


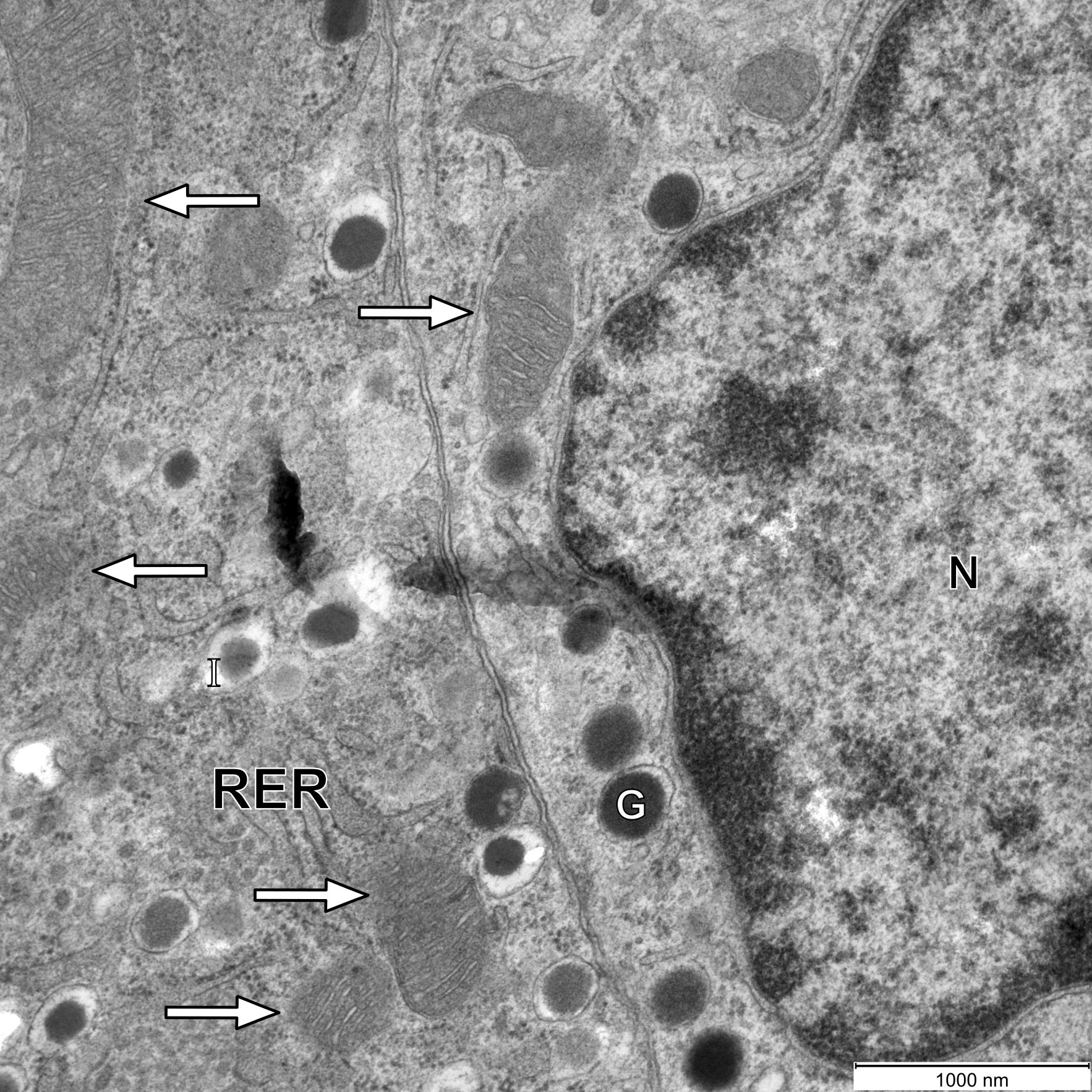


**Fig. S11** Ultrastructure of an α- and a $\beta$-cell in a control mouse. The mitochondria in both types of cells are elongated, oval, showing the typical inner organization. The outer and inner membranes are well seen and the inner membrane is organized in characteristic cristae. G, glucagon granule; I, insulin granule; N, nucleus; RER, rough endoplasmic reticulum; the white arrows point on mitochondria. Scale bar: 1000 nm.


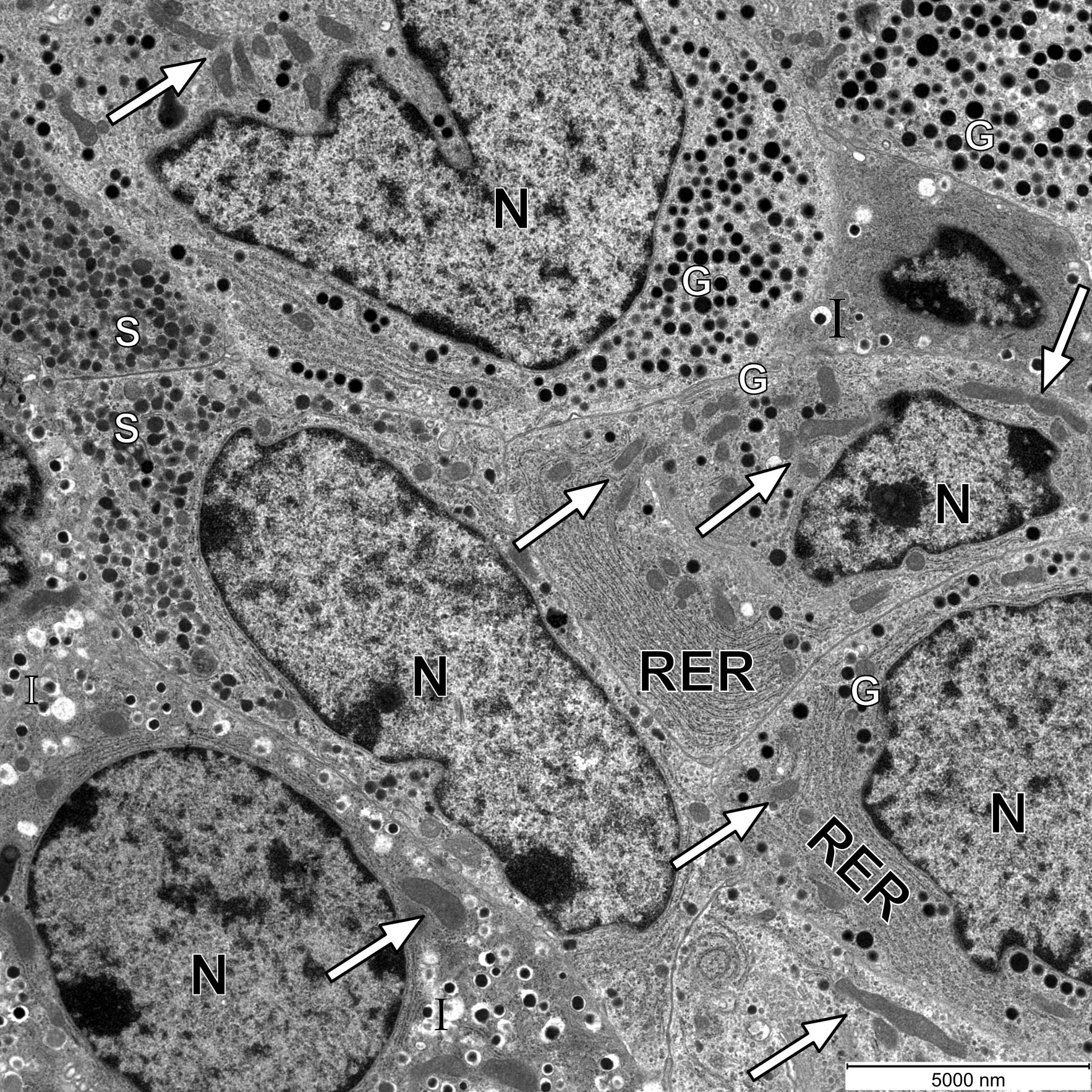


**Fig. S12** Ultrastructure of an α-, a $\beta$-, and a $\delta$-cell in a control mouse. The majority of mitochondria in all types of cells are elongated, oval, showing the typical inner organization. The outer and inner membranes are well seen and the inner membrane is organized in characteristic cristae. G, glucagon granule; I, insulin granule; S, somatostatin granule; N, nucleus; RER, rough endoplasmic reticulum; the white arrows point on mitochondria. Scale bar: 5000 nm.

**S2 Sensitivity analysis of the mathematical model**

In order to evaluate the performance of our mathematical model, we analysed its sensitivity to changes in selected parameters. Specific modules incorporated in our model have been examined before. A detailed analysis of the mathematical model simulating α-cell electrical and Ca^2+^ activity as well as the corresponding glucagon secretion has been published in [1,2]. Furthermore, the behaviour of the mathematical model for glycolysis and glucose driven mitochondrial activity has also been studied in detail [3]. Therefore, we are here focusing on the parameters that influence the coupling between the modules and the additionally introduced elements of the model. In accordance with the structure of our model, we split the sensitivity analysis for particular parameters into three subsections, reflecting the three main processes studied here: 1) Glucose uptake and glycolysis, 2) Glucose and free fatty acid oxidation, and 3) Glucagon secretion. We use a variant of the one-at-a-time (OAT) method to perform the sensitivity analysis [4]. Results of our sensitivity analysis are presented graphically and show how a relative change of a selected parameter affects the relative glucagon secretion (RGS). In our investigation, we focused on both low glucose (G = 1 mM) and high glucose (G = 6 mM) conditions.

## S2.1 Glucose uptake and glycolysis

The mathematical formalism for glucose transport into the cell is given by Eq. (1) in the manuscript. We link the Eq. (1) with the glycolytic part of the model that is based on the theoretical framework proposed by Smolen [3]. Here, the parameters *V*_max,GK_ and *K*_m,GK_ are of pivotal importance. *V*_max,GK_ is the maximal reaction rate and *K*_m,GK_ is the corresponding half-saturation constant. These two parameters determine the flux *J*_GK_ in dependence on the glucose concentration (*G*), which in turn affects glucagon secretion.

Results presented in Fig. S13 show that at low glucose concentration (*G* = 1 mM), variations of *V*_max,GK_ and *K*_m,GK_ have practically no effect on glucagon secretion. At a higher glucose level, i.e., *G* = 6 mM, however, a decrease in the *V*_max,GK_ causes an increase in glucagon secretion, and vice versa. If *V*_max,GK_ is reduced, less glucose enters the cell (accordingly to Eq. (1)), thereby reducing the ATP concentration and RAT, and increasing K_ATP_-channel conductivity (Eq. (16)). In turn, this leads to an increased glucagon secretion. Higher values of the half-saturation constant *K*_m,GK_ cause a larger glucagon secretion because of the increased glucose transport into the cell. Fig. S13 therefore shows that the model is robust to variations in the parameters *V*_max,GK_ and *K*_m,GK_, e.g., when the parameters are changed by up to 20 %, glucagon secretion at *G* = 1 mM is always higher than at *G* = 6 mM, which realistically reflects the physiological conditions in α-cells.


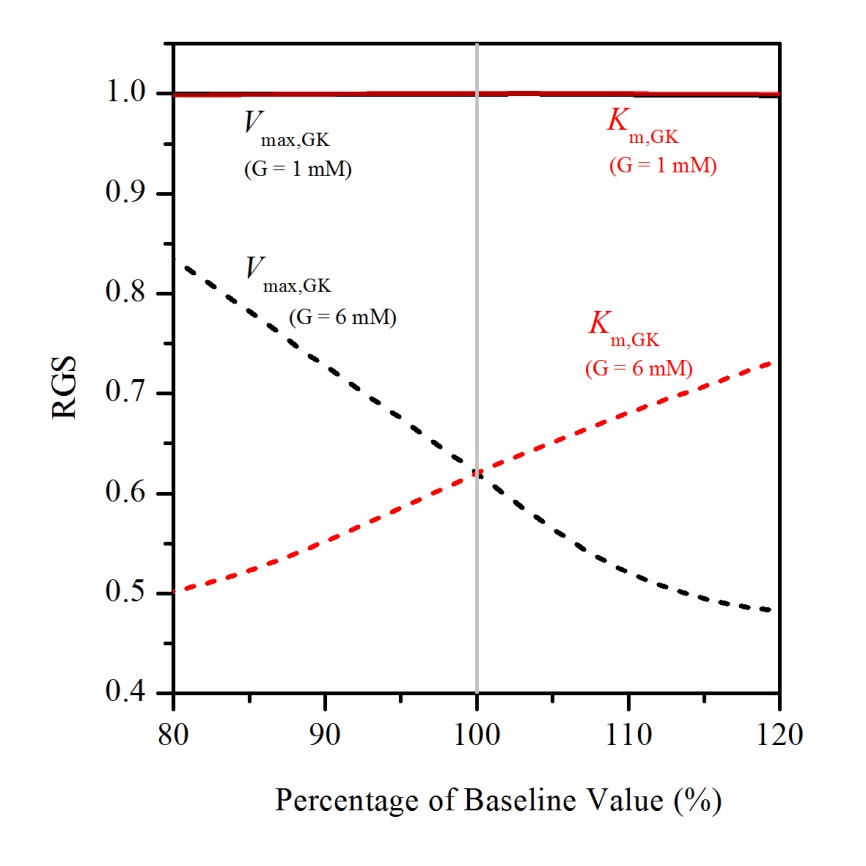


**Fig. S13** Sensitivity of the relative glucagon secretion (RGS) to model parameters: *V*_max,GK_, the maximum reaction rate (black lines) and *K*_m,GK_, the half-saturation constant (red lines), at two different glucose concentrations: *G* = 1 mM (solid lines) and *G* = 6 mM (dashed lines). The baseline values: *V*_max,GK_ = 1 10^-3^ μM/ms, *K*_m,GK_ = 8.5 mM.

## S2.2 Glucose and free fatty acid oxidation

In the model, the glucose and the FFAs are considered as energy sources for mitochondrial ATP production (Eqs. 7-12). The main parameter in the model influencing the glucose oxidation is *k*_GO_. This parameter represents the net yield of ATP per glucose molecule (Eq. 7). The oxidation of FFA is fine-tuned with the parameter *k*_FFAO_, representing the FFA oxidation rate constant (Eq. 8). The Randle cycle is also included via the parameter *k*_R_ (Eq. 8). In Eqs. (7) and (8), the parameter *k*_md_ is introduced for modelling the level of mitochondrial dysfunction. The sensitivity analysis of these parameters is presented in Fig. S14.

The relative changes in parameters *k*_GO_ and *k*_FFAO_ have a proportional effect on the relative changes in fluxes *J*_GO_ (Eq. 7) and *J*_FFAO_ (Eq. 8). Fig. S14 shows that glucagon secretion is more sensitive to changes of the parameter *k*_FFAO_ then to changes of the parameter *k*_GO_. The latter can be explained by the fact that in α-cells the oxidation of FFAs (*J*_FFAO_) is higher than the oxidation of glucose products (*J*_GO_), especially at low glucose concentrations (*G* = 1 mM), when changes in *k*_GO_ do not really affect the RGS at all. It should be noted that the bell-shaped response with a clear maximum around the baseline value, inferred by variations of *k*_FFAO_, is a consequence of the core alpha cell model structure, which predicts the maximum secretion rate at 1 mM glucose. Consequently, any kind of variations cause a decrease in RGS. At low glucose (*G* = 1 mM), the RGS is also not affected by changes in the parameter *k*_R_, whereas at higher glucose (*G* = 6 mM) it only has a moderate effect on the RGS.

A 10 % decrease in the *k*_FFAO_ increases the RGS that becomes even higher at *G* = 6 mM than at *G* = 1 mM (see Fig. S14). This is in line with the predictions of our model (Fig. 4c), where we show that mitochondrial dysfunction leads to the same effect. Namely, the reduction of *k*_FFAO_, in terms of ATP production, plays a similar role as the mitochondrial dysfunction. From Eqs. (7) and (8) it can be seen that changes in the parameters *k*_GO_ and *k*_FFAO_ have the same effect on changing the currents *J*_GO_ and *J*_FFAO_ as the factor (1-*k*_md_), where the *k*_md_ represents the level of mitochondrial dysfunction. This is also evident from the comparison of the effects of the *k*_FFAO_ (Fig. S14) and the factor (1-*k*_md_) (Fig. S15), where we see that the parameter changes have similar effects on the RGS. The system is slightly more sensitive to changes of *k*_FFAO_ than to changes in (1-*k*_md_). This is due to the fact that *k*_md_ also appears in Eq. (10) and mimics the corresponding ATPases reduction (*k*_ATPase,r_), while this is not taken into account when changing the *k*_FFAO_. The ATPases have a great impact on cytosolic ATP concertation and, consequently, on the RGS.

**
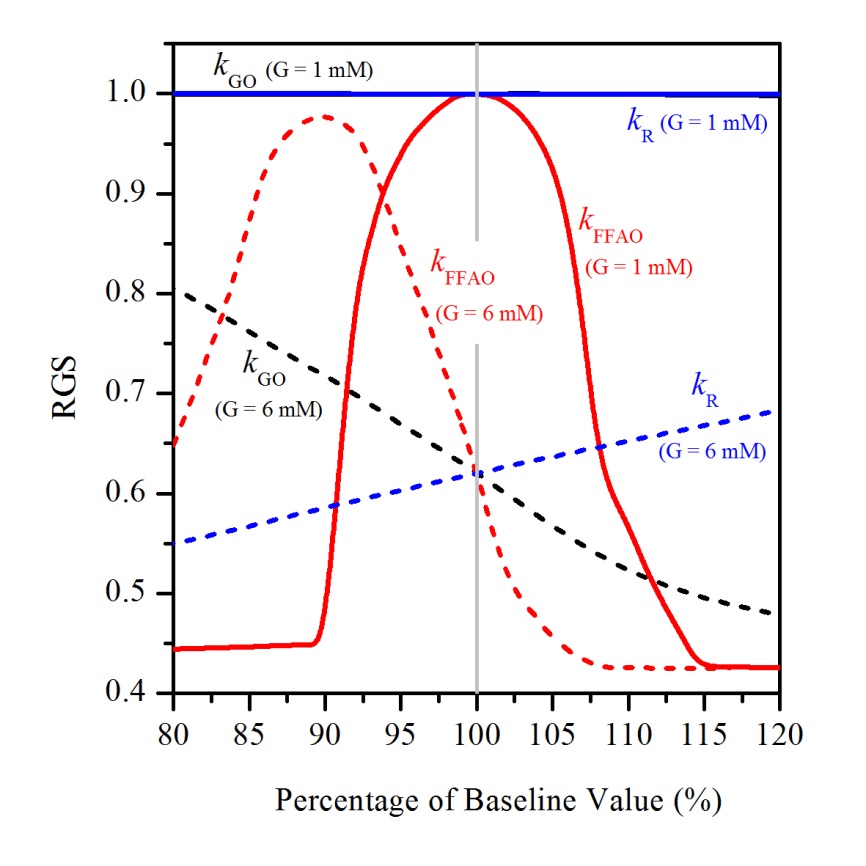
**

**Fig. S14** Sensitivity of the relative glucagon secretion (RGS) to model parameters: *k*_GO_, the net yield of ATP per glucose molecule (black lines); *k*_FFAO_, the FFA oxidation rate constant (red lines); *k*_R_, the glucose reduction factor representing the effect of the Randle cycle (blue lines), at two different glucose concentrations: *G* = 1 mM (solid lines) and *G* = 6 mM (dashed lines). The baseline values: *k*_GO_ = 38/2, *k*_FFAO_ = 0.1 μMms^-1^, *k*_R_ = 0.01 mM^-1^.

**
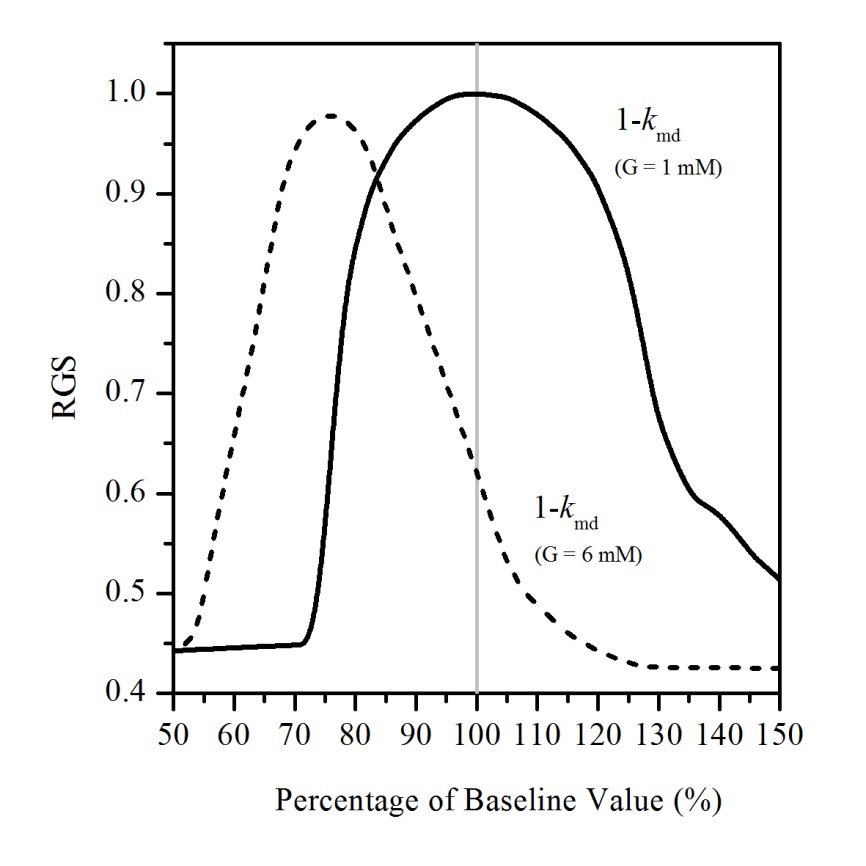
**

**Fig. S15** Sensitivity of the relative glucagon secretion (RGS) to the model parameter *k*_md_ representing the level of mitochondrial dysfunction (e.g., *k*_md_ = 0.3 reflects a 30 % decrease in mitochondrial function). For physiological conditions without mitochondrial dysfunction it holds 1-*k*_md_ = 1. The sensitivity analysis is provided for two glucose concentrations: *G* = 1 mM (solid line) and *G* = 6 mM (dashed line).

The sensitivity of the model predictions for RGS in dependence on the activity of the ATPases is shown in Fig. S16. We can see that the mathematical model expresses a similar sensitivity to changes in parameters *k*_ATPase_ and *k*_FFAO_ (see Fig. S14). Namely, the parameters determine the magnitudes of the energy fluxes *J*_FFAO_ and *J*_ATPase_, which are comparable but significantly larger than the other fluxes in Eq. (11). The parameters *k*_ATPase_ and *k*_FFAO_ have therefore a decisive influence on the size of the cytosolic ATP concertation. This makes the model most sensitive to these two parameters, except that a decrease in *k*_FFAO_ (Eq. (8)) has the same effect as an increase in *k*_ATPase_ (Eq. (10)), which is also evident from the comparison of the sensitivity for parameters in Figs. S14 and S16.

Lastly, we analysed the impact of variations of the parameters in Eq. (10) used to model the reduction of ATP production due to mitochondrial dysfunction (*k*_md_) along with the simultaneous ATPase reduction *k*_ATPase,r_. Results presented in Fig. S16 show that the *k*_md_ governs the behaviour of the model, as it acts as a factor in Eq. (10). For example, at a 30 % decrease in mitochondrial function (*k*_md_ = 0.3), the sensitivity of the model to the parameter *k*_ATPase,r_ is not as large as to the parameter *k*_ATPase,r_ and under conditions with the normal mitochondrial function (*k*_md_ = 0), changes in *k*_ATPase,r_ do not affect RGS at all. We can also see that an increase in *k*_ATPase,r_ at low glucose concentration (*G* = 1 mM) and the 30 % mitochondrial dysfunction leads to an increased RGS, which approaches the physiological level for normal mitochondrial functioning. This is due to the fact that a reduced activity of the ATPases leads to an increase in the cytosolic ATP concentration, and represents a kind of a compensatory effect to the lower ATP production by less efficient mitochondria.


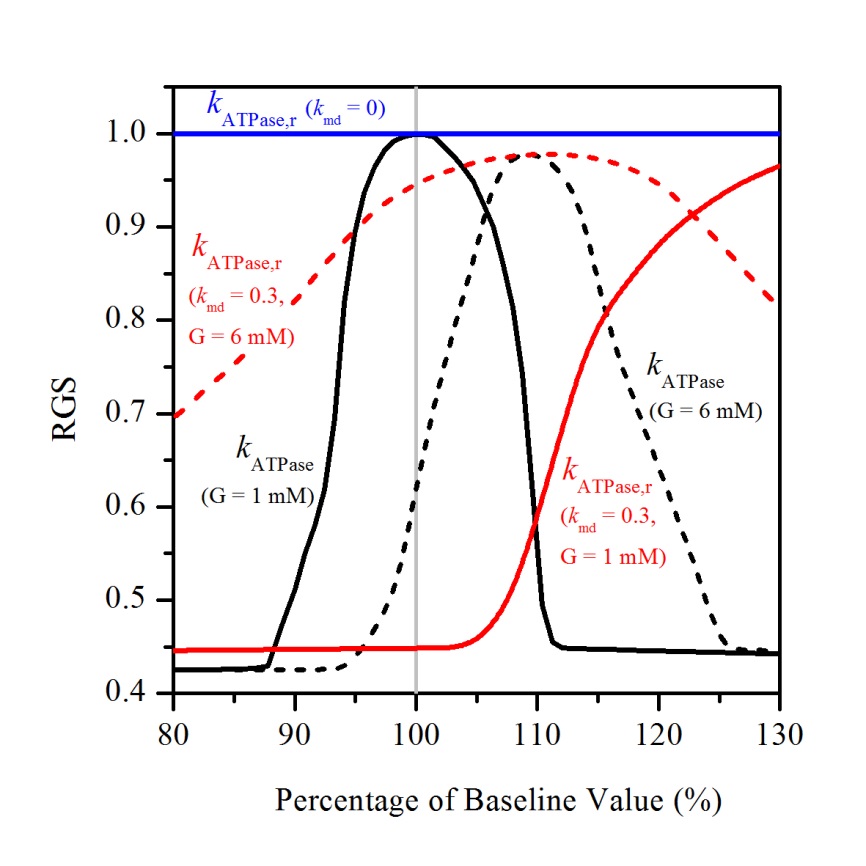


**Fig. S16** Sensitivity of the relative glucagon secretion (RGS) to model parameters: *k*_ATPase_, the rate constant of ATPases (black lines); *k*_ATPase,r_, the ATPases reduction for the corresponding mitochondrial dysfunction *k*_md_ = 0.3 (red lines) and *k*_md_ = 0 (blue lines), at two glucose concentrations: *G* = 1 mM (solid lines) and *G* = 6 mM (dashed lines). The baseline values: *k*_ATPase_ = 5^.^10^-5^ ms^-1^, *k*_ATPase,r_ = 0.7.

## S2.3 Glucagon secretion

Here we make the sensitivity analysis for parameters directly linking the metabolic processes in the cell with glucagon secretion. As mentioned earlier, we do not perform a sensitivity analysis for the parameters that govern α-cell glucagon secretion and are part of a previously published mathematical models [2,5]. In terms of the robustness of our model, we focus on the coupling between the ATP/ADP ratio (Eq. (14)) and the K_ATP_-channel conductance (Eq. (16)). The parameters *g*_1_ and *k*_g, KATP_ in Eq. (16), that were determined by fitting an exponential function to the experimentally measured values, play a pivotal role. The sensitivity of the model to these two parameters is shown in Fig. S17. It can be seen that at *G* = 1 mM, the system is slightly more sensitive to the parameter *g*_1_ than to the *k*_g, KATP_. At *G* = 6 mM, however, the sensitivity of both parameters is approximately the same. If we decrease the parameter *g*_1_, we get a similar response in the RGS as by increasing the parameter *k*_g, KATP_, and vice versa. Under physiological conditions, without mitochondrial dysfunction, a change in *k*_g, KATP_ within 20 % still indicates greater glucagon secretion at *G* = 1 mM than at *G* = 6 mM. Due to cascade mechanisms, the model also considers the smoothing of the oscillating variation of RAT, where the parameter *k*_f_ regulates the level of the smoothing (Eq. 15). Fig. S17 shows that the system is robust with respect to changes of this parameter.


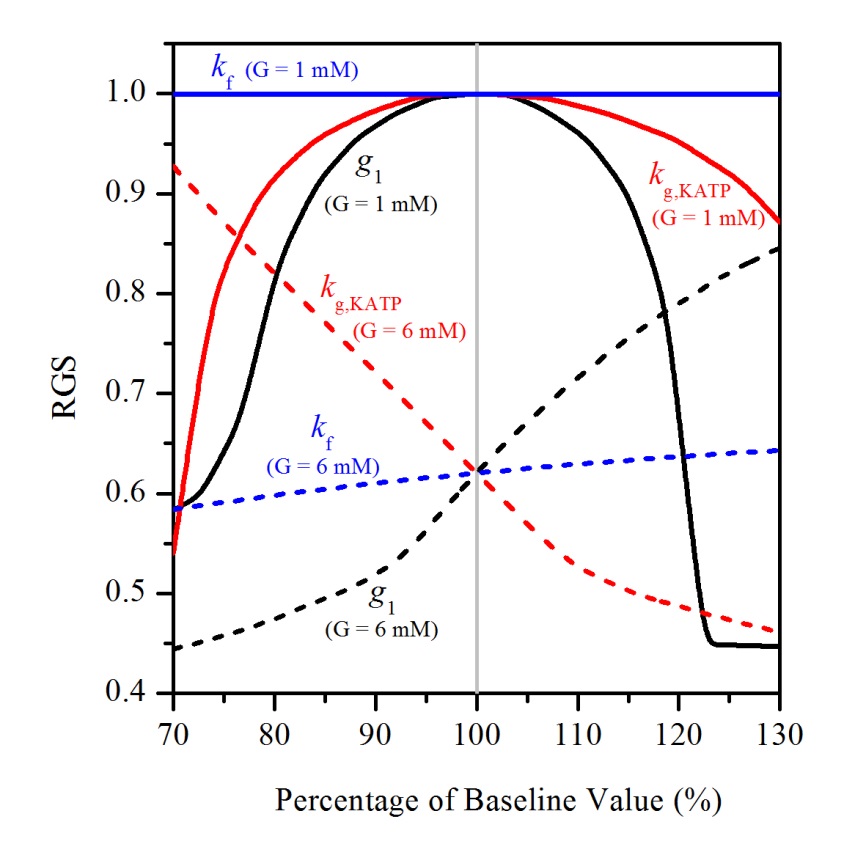


**Fig. S17** Sensitivity of the relative glucagon secretion (RGS) to model parameters: *g*_1_ (black lines), *k*_g,KATP_ (red lines), and *k*_f_ = (blue lines), at two different glucose concentrations: *G* = 1 mM (solid lines) and *G* = 6 mM (dashed lines). The baseline values: *g*_1_ = 0.5 nS, *k*_g,KATP_ = 0.2, *k*_f_ = 0.01 s^-1^.

In summary, the results of the sensitivity analysis show that the model robustly predicts glucagon secretion with respect to variations in model parameters. The sensitivity analysis reflects the robustness of the model predictions for a broader range of parameter changes, in which the model predictions remain in agreement with those obtained for the reference set of model parameters (Tables 1-3). Moreover, this analysis provides a deeper insight into the whole parameter space revealing the decisive parameters that considerably affect the energetic processes in the cell and the related glucagon secretion. This represents an important additional insight for a better understanding of the bioenergetic disruptions and the mitochondrial dysfunction.

# References

1. Watts M, Sherman A. 2014 Modeling the pancreatic α-cell: Dual mechanisms of glucose suppression of glucagon secretion. *Biophys. J.* **106**, 741–751. (doi:10.1016/j.bpj.2013.11.4504)

2. Montefusco F, Pedersen MG. 2015 Mathematical modelling of local calcium and regulated exocytosis during inhibition and stimulation of glucagon secretion from pancreatic alpha‐cells. *J. Physiol.* **593**, 4519–4530. (doi:10.1113/JP270777)

3. Smolen P. 1995 A model for glycolytic oscillations based on skeletal muscle phosphofructokinase kinetics. *J. Theor. Biol.* **174**, 137–148. (doi:10.1006/jtbi.1995.0087)

4. Link KG, Stobb MT, Di Paola J, Neeves KB, Fogelson AL, Sindi SS, Leiderman K. 2018 A local and global sensitivity analysis of a mathematical model of coagulation and platelet deposition under flow. *PLoS One* **13**, e0200917. (doi:10.1371/journal.pone.0200917)

5. Quesada I, Tudurí E, Ripoll C, Nadal Á́. 2008 Physiology of the pancreatic α-cell and glucagon secretion: Role in glucose homeostasis and diabetes. *J. Endocrinol.* **199**, 5–19. (doi:10.1677/JOE-08-0290)
